# The effectiveness of immersive virtual reality in physical recovery of stroke patients: A systematic review

**DOI:** 10.3389/fnsys.2022.880447

**Published:** 2022-09-22

**Authors:** Irini Patsaki, Nefeli Dimitriadi, Akylina Despoti, Dimitra Tzoumi, Nikolaos Leventakis, Georgia Roussou, Argyro Papathanasiou, Serafeim Nanas, Eleftherios Karatzanos

**Affiliations:** ^1^Clinical Ergospirometry, Exercise and Rehabilitation Laboratory, 1st Critical Care Department, Evangelismos Hospital, School of Medicine, National and Kapodistrian University of Athens, Athens, Greece; ^2^Department of Film Studies, Aristotle University of Thessaloniki, Thessaloniki, Greece; ^3^Virtual Reality Applications (ViRA), Athens, Greece

**Keywords:** virtual reality, immersive, stroke, physical rehabilitation, functional ability

## Abstract

**Background:**

Over the past few years, technological innovations have been increasingly employed to augment the rehabilitation of stroke patients. Virtual reality (VR) has gained attention through its ability to deliver a customized training session and to increase patients’ engagement. Virtual reality rehabilitation programs allow the patient to perform a therapeutic program tailored to his/her needs while interacting with a computer-simulated environment.

**Purpose:**

This study aims to investigate the effectiveness of a fully immersive rehabilitation program using a commercially available head-mounted display in stroke patients.

**Methods:**

A systematic search was conducted in three databases, namely, PubMed, Google Scholar, and PEDro. Four hundred thirty-two references were identified. The keywords used for the literature search were in English, which are given as follows: immersive, virtual reality, neurorehabilitation, stroke, and head-mounted display. Additionally, applicable articles were identified through screening reference lists of relevant articles.

**Results:**

Only 12 studies used head-mounted display for immersing the patient into the virtual world. Apart from the feasibility of this new technology, a range of benefits were identified, especially in terms of functional ability as measured by FIM or Barthel, the Action Research arm Test, Box and Block Test, Fugl-Meyer assessment of physical performance, strength, and balance outcomes.

**Conclusion:**

The results from this review support the potential beneficial effect of fully immersive virtual reality in the rehabilitation of stroke patients, maximizing recovery through increased motivation and adherence.

## Introduction

Stroke is a neurological disease that has been described as a leading cause of disability with substantial economic cost for rehabilitation (Gbd 2016 Stroke Collaborators, 2019). Stroke survivors face a wide range of disabilities from cognition to paresis that led to reduced functional ability and quite often to loss of independence. Functional decline is apparent even 5 years post-stroke, leading to long-term deficits, especially regarding activities of daily living (Dhamoon et al., 2009).

There is high-quality evidence that supports the use of rehabilitation intervention to improve physical functioning even in severe stroke survivors (McGlinchey et al., 2020). Different rehabilitation techniques have been used to accelerate recovery. All of them have induced substantial beneficial effects. Technological innovations have offered severe advantages in facing the complicity of stroke rehabilitation. Virtual reality (VR) interventions have been used the past few years as a therapeutic means to improve physical recovery through enhancing neuronal plasticity and relearning movement patterns and motor skills (Raffin and Hummel, 2018). Additionally, the gamification of rehabilitation has increased motivation and adherence, both of which are key aspects of a successful recovery (Dias et al., 2019). It is believed that VR has the means to incorporate key elements in the rehabilitation program such as motor-cognitive training, different neuroscience principles, motivational game themes, and empowerment techniques and advance the whole process of recovery (Perez-Marcos et al., 2018). Immersion is a vital component of the whole experience and is a key component to the feeling of presence. Although full immersion in the virtual environment enhances the experience and strengthens the engagement of the patient, it seems to face certain challenges regarding the inclusion of haptic devices (Rose et al., 2018). The term “virtual reality” has been excessively used, but often describes different systems from display monitors to head-mounted displays (HMD). In a recent study, Huygelier et al. (2021) tried to underline the differences between mixed reality systems and virtual reality, in an effort to distinguish to what extent virtual information and real information are mixed. The term “fully immersive” environment presupposes stereoscopic vision and the complete disconnection of the user from his physical environment. Another term frequently used under the umbrella of VR is that of augmented reality. In this study, virtual information is superimposed over the real world. Different terminologies could create further confusion in relation to the degree of immersion being achieved, yet this could be overcome by looking into the input devices being used. It is well noted by Huygelier et al. (2021) that it is important to start using a consistent terminology regarding VR technologies as there is a continuous evolution in the field. Systematic reviews that have been published examining the effectiveness of virtual reality have included both fully and semi-immersive studies, in which different types of visualization devices have been used such as PC (desktop VR) and TV monitor. Over the past decades, technological innovation has included from commercial gaming systems to custom-made specific VR (Huygelier et al., 2021).

The objective of this systematic review was to investigate the effectiveness of a fully immersive VR rehabilitation program using a head-mounted display on upper limb function, gait, and balance in stroke patients.

## Methods

The current review was conducted following the preferred reporting items for systematic reviews and meta-analyses (PRISMA) statement. The protocol was not registered.

### Searching strategy

The electronic databases of PubMed, PEDro, and Google Scholar were systematically searched for studies published till January 2021. Additionally, we thoroughly examined the reference lists from the studies included to identify further relevant studies. The keywords used for this systematic review were “virtual reality,” “immersive,” “stroke,” “rehabilitation,” “Recovery,” “Oculus,” and “Head Mounted Display,” These were used with certain combinations such as [(immersive virtual reality) OR (Head Mounted Display)] AND [(stroke rehabilitation) OR (stroke recovery)]. The search was performed by two of the authors who additionally screened the titles and the abstracts for inclusion.

### Eligibility criteria and study selection

Only randomized control studies with fully immersive VR technologies (head-mounted display) that were published in English in peer-reviewed journals and measured rehabilitation in physical functions (muscle strength, activities of daily living, functional ability, gait parameters, and balance) after stroke were included. The studies with psychiatric disorders and those that assessed only cognitive outcomes were excluded. Studies that provided only neurocognitive rehabilitation were also excluded.

### Methodological quality

The quality of the included studies was evaluated by the Physiotherapy Evidence Database (PEDro) Scale. Total scores from 6 to 10 were considered high quality, 4 to 5 were considered fair quality, and ≤3 were considered poor quality. Two authors conducted a blinded rating of the methodological quality of the studies. Different rates and unclear issues were discussed.

## Results

### Selection of studies

**FIGURE 1:**
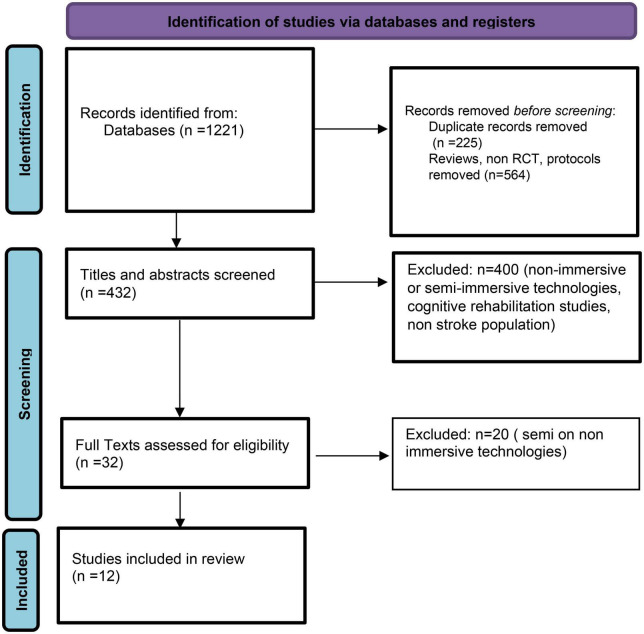
Flow diagram of selected studies being included in the systematic review.

From the initial search, 1,221 articles were identified. Non-RCT, cases of series, protocols, and duplicates were excluded. Four hundred thirty-two articles were remained to be investigated regarding the type of immersion used. After screening the titles, the abstracts and in a few cases the whole article for eligibility criteria, 12 studies (Table 1) were selected to be included in this systematic review. The selection process is summarized according to PRISMA guidelines as a flowchart (Figure 1).

**TABLE 1:** Characteristics of all included studies.

Study	Patients	Equipment used besides VR	Control group	Training task	Outcome	Results
Jaffe et al., 2004	20 (EG:10/CG:10) Chronic stroke	Treadmill	Conventional training with foam made obstacles	Gait	Walk speed, stride length, step length, 6 MWT	Intervention had a significant faster walking speed and longer stride length for the fast pace walking (*p* < 0.01)
Ma and Bechkoum, 2008	8 (EG:4/CG:4) Chronic stroke	Controllers	Functional training	Arm rehab	ARAT, Motricity Index	The VR intervention presented a higher probability for improvement regarding the MI (*p* = 0.0389 vs. *p* = 0.1391), similar results were noted in ARAT too
Connelly et al., 2010	14 (EG:7/CG:7) Chronic stroke	PneuGlove	Same training outside the VR environment	Arm	FMA-UE, box blocks T, grip strength, lateral, and palmar pinch	No significant difference between groups (*p* = 0.904)
Crosbie et al., 2012	18 (EG:9/CG:9) Chronic stroke	Sensors	Physical therapy	Arm	ARAT, Motricity Index	No statistical significance differences between groups (MI: *p* = 0.485, ARAT: *p* = 0.139)
Jung et al., 2012	21 (EG:11/CG:10) Chronic stroke	Treadmill	Conventional treadmill training	Balance	TUG, ABC scale	Significant difference between groups TUG (-2.7 ± 1.9 vs. -0.8 ± 0.7, *p* < 0.05) ABC (9.5 ± 6.0 vs. 4.3 ± 3.3, *p* < 0.05)
Kang et al., 2012	30 (EG:10/TIG:10/CG:10) Chronic stroke	Treadmill with optic flow	TIG: Conventional treadmill CG: physical therapy	Gait/balance	TUG, 10 MWT, 6 MWT, FRT	Significant difference between groups TUG (13.2 ± 2 vs. 17.9 ± 4.5 vs. 20 ± 5.0, *p* < 0.001) FRT (30.7 ± 1.3 vs. 30.4 ± 2.5 vs. 28.2 ± 2.3, *p* < 0.001) 6 MWT (264.8 ± 18.6 vs. 242.3 ± 26.0 vs. 240.9 ± 22.4, *p* < 0.001)
Kim and Lee, 2012	19 (EG:10/CG:09) Chronic stroke (>6 months)	Treadmill + FES	Treadmill + FES	Balance Gait	TUG, BBS	Significant difference between groups: TUG (-7.54 ± 2.74 vs. -6.14 ± 2.57, *p* < 0.05)
Park et al., 2013	16 (EG:8/CG:8) Chronic stroke		Physical therapy	Gait	Velocity, cadence, step length, stride length, 10 MWT	Significant difference between groups only in stride length (*p* < 0.05)
Lee et al., 2014	21 (EG:10/CG:11) Chronic stroke (>6 months)		Physical therapy	Posture (balance/gait)	TUG, BBS, velocity, cadence, step, and stride length	No difference between groups
Ögün et al., 2019	64 (EG:32/CG:32) Chronic stroke	Leap motion tracking device	Conventional upper extremity exercises	Arm rehab	ARAT, FIM, FMA-UE, PASS	Significant difference (*p* < 0.001) between groups for all outcomes ARAT (8.33 ± 4.44 vs. 1.25 ± 1.45) FMA-UE (6.90 ± 3.99 vs. 1.50 ± 1.48)
Cho and Lee, 2019	42 (EG:21/CG:21) Acute stage		Computerized cognitive therapy	Arm	FIM	Significant difference between groups in functional independence measure (19.19 ± 13.2 vs. 9.43 ± 15, *p* < 0.05)
Mekbib et al., 2021	23 (EG:12/CG:11) Sub-acute (<3 months)	Leap motion tracking device	Occupational Therapy	Arm rehab	FMA-UE, BI	Significant difference between groups in FMA-UE (12.25 ± 4.58 vs. 7.704 ± 2.54, *p* = 0.007).

CS, case study; EG, experimental group; TIG, traditional intervention group; CG, control group; CybGlov, cyber glove; ARAT, action reach arm test; FMA-UE, Fugl-Meyer assessment of upper extremity function; ABC, activities balance confidence; BBS, Berg Balance Scale; BI, Barthel Index; FRT, functional reach test; FIM, functional independence measurement; PPT, Purdue Pegboard test; TUG, time up and go.

### Methodological quality

All studies included were assessed for methodological quality using the PEDro Scale (Table 2). Five studies (Crosbie et al., 2012; Kang et al., 2012; Lee et al., 2014; Ögün et al., 2019; Mekbib et al., 2021) were considered “high quality” and seven studies (Jaffe et al., 2004; Ma and Bechkoum, 2008; Connelly et al., 2010; Jung et al., 2012; Kim and Lee, 2012; Park et al., 2013; Cho and Lee, 2019) were considered “moderate quality.” The mean score of the methodological quality for the included studies was 5.67.

**TABLE 2:** Quality of the RCT studies of stroke patients on PEDro Scale (item 1 does not contribute to total score).

	1	2	3	4	5	6	7	8	9	10	11	
Jaffe et al., 2004	*	*		*				*			*	4/10
Ma and Bechkoum, 2008				*	*			*		*		4/10
Connelly et al., 2010	*	*		*				*		*		4/10
Crosbie et al., 2012	*	*	*	*			*	*	*	*	*	8/10
Jung et al., 2012	*	*		*			*			*	*	5/10
Kang et al., 2012	*	*	*	*			*	*		*	*	7/10
Kim and Lee, 2012	*	*		*						*	*	4/10
Park et al., 2013	*	*		*				*		*	*	5/10
Lee et al., 2014		*		*			*	*	*	*	*	7/10
Ögün et al., 2019	*	*	*	*	*		*			*	*	7/10
Cho and Lee, 2019	*	*		*	*			*		*	*	6/10
Mekbib et al., 2021	*	*	*	*			*	*		*	*	7/10

The * represents the items that were scored.

### Type of virtual environment and gamification scenarios

Full immersion of the patient in the training environment was achieved through a head-mounted display (HMD). This equipment displays the virtual environment and completely isolates patients from the surrounding environment, providing a better sense of presence (Radianti et al., 2020). Additionally, a series of input devices such as a real-time motion tracking devices were used offering a more augmented environment or different kinds of magnets were used to track motion (Ma and Bechkoum, 2008; Connelly et al., 2010). In recent years, devices such as the leap motion have been introduced to provide a sense of crabbing an object without employing a different device to track the motion (Ögün et al., 2019; Mekbib et al., 2021). In terms of upper limb rehabilitation, different special gloves (Connelly et al., 2010; Crosbie et al., 2012) were used to facilitate training, while in terms of gait, a treadmill (Jung et al., 2012; Kang et al., 2012; Kim and Lee, 2012) was often used. It is well-established that all studies offer a different degree of intrinsic feedback, with all having visual and auditory and some somatosensory ones. Extrinsic feedback, in the form of grading one’s performance, was an important feature that facilitates the objective progression of the task while keeping the patient motivated and interested. Progression often had to do with increasing walking distance or the distance from the handling object or even decreasing the time needed to perform the task.

The studies included in the review made a significant effort to enhance motor learning principles as seen in more conventional rehabilitation strategies. In most of the studies, information regarding progression, use of different tasks, and task specificity are well-described. Task variations regarding balance and gait rehabilitation involved obstacle negotiation (Jaffe et al., 2004) and treadmill training (Connelly et al., 2010; Jung et al., 2012; Kang et al., 2012). Through the HMD, the patients experienced a normal view next to their own while walking in a simulated park or street. This visual feedback acted as a continuous stimulus to correct one’s movement. All subjects who used a treadmill wore a suspension device for safety purposes. Treadmill with the optic flow program (Kang et al., 2012) employed computer hardware for the output and was applied to the HMD. Speed, controlled through the optic flow, was continuously increased. Jaffe et al. used a park simulation presented through the HMD device. Upper extremity rehabilitation included picking up, reaching, moving different objects, and dual-task training. Different scenarios were used such as: decorating a tree with leaves and fruits or picking up vegetables from a bowl and putting them back (Ögün et al., 2019), a drumming game (Ögün et al., 2019), catching fruits that fall off a tree with a basket (Ma and Bechkoum, 2008), fishing games, or hitting mice with a virtual hammer (Ma and Bechkoum, 2008; Crosbie et al., 2012).

### Upper limb functionality

Five studies aimed to assess the effectiveness of a fully immersive program on arm’s functional recovery. It was noted that three studies (Ma and Bechkoum, 2008; Ögün et al., 2019; Mekbib et al., 2021) pointed out the significant difference between groups in favor of the VR program. While the other two (Connelly et al., 2010; Crosbie et al., 2012) didn’t reach statistical significance, they pointed out that the VR intervention was more effective. Muscle strength was assessed only in two studies (Connelly et al., 2010; Kim and Lee, 2012). In terms of upper limb rehabilitation, it was demonstrated a significant improvement over time in the palmar pinch, which is used in all daily activities that include picking up and releasing objects. In this study, the use of a pneumo-glove was essential for this measurement. In terms of gait rehabilitation, Kim and Lee (2012) reported a significant change in the tibialis anterior and quadriceps femoris muscles of the affected side when VR was used along with functional electrical stimulation.

The instruments that were mostly used were the Fugl-Meyer Upper Extremity (FM-UE) function (Connelly et al., 2010; Ögün et al., 2019; Mekbib et al., 2021) and the Action Reach Arm Test (ARAT) (Ma and Bechkoum, 2008; Crosbie et al., 2012; Ögün et al., 2019). Additionally, the Motricity Index (Ma and Bechkoum, 2008; Crosbie et al., 2012); the Box Block Test (Connelly et al., 2010); and muscle strength by measuring grip strength, lateral pinch, and palmar pinch strength (Connelly et al., 2010) were used.

### Functional ability

Three studies (Ögün et al., 2019; Mekbib et al., 2021; Cho and Lee, 2019) examined the improvement of functional ability and used the functional independence measurement (FIM), Barthel Index (BI), and performance assessment of self-care skills (PASS). All instruments have been developed to assess an individual’s independence in performing activities of daily living. All studies found statistically significant improvement of the VR intervention in relation to the control.

### Balance-gait

Balance was examined in four studies by the Time-Up and Go Test (TUG) (Jung et al., 2012; Kim and Lee, 2012; Park et al., 2013; Lee et al., 2014). The test was significantly improved in three of the studies (Jung et al., 2012; Kang et al., 2012; Kim and Lee, 2012), while in only one study (Lee et al., 2014), the intervention group showed better results without reaching statistical significance. Berg Balance Scale (BBS) was also used in two studies (Kim and Lee, 2012; Kim and Lee, 2012) with improvement in the intervention group although present not being able to reach statistical significance again.

Gait (Jaffe et al., 2004; Kang et al., 2012; Park et al., 2013; Lee et al., 2014) was assessed by numerous parameters such as velocity, cadence, step length, and stride length. Endurance was examined either by 6 MWT (Jaffe et al., 2004; Kang et al., 2012) or by 10 MWT (Kang et al., 2012; Lee et al., 2014). Patients demonstrated clinically meaningful changes in the gait parameters in all training groups, and in some of them, there was a statistically significant difference in favor of the experimental group.

#### Safety side effects

Only two studies examined the appearance of any side effects from the immersion into virtual reality (Jaffe et al., 2004; Crosbie et al., 2012). Jaffe et al. (2004) reported no incidence of falling due to the dynamic nature of the exercise, and Crosbie et al. (2012) mentioned only the appearance of dizziness in two patients.

## Discussion

The purpose of this systematic review was to assess the effectiveness of a fully immersive virtual reality rehabilitation program using a head-mounted display on upper limb function, gait, and balance in stroke patients. As we were interested in the physical interaction of the user within the digital environments, and to assess its effectiveness, the focus on fully immersive technologies was most appropriate. Thus, in this systematic review, we included only fully immersive studies. Although we noted a variety regarding the haptic devices used in studies that aimed at arm’s functional rehabilitation and the duration of the programs in general, the results suggested a positive impact of the immersive VR applications on upper extremity function, gait, and balance in stroke patients. Even in more dynamic activities such as walking, VR interventions were safely delivered with positive effects on velocity and step-stride length. Jaffe et al. (2004) stated that patients did not mention any dizziness even one that suffered claustrophobia episodes at home. Thus, VR interventions could also involve both dynamic and stationary activities. When VR was used in upper limb rehabilitation, only two patients reported dizziness and headache (Crosbie et al., 2012). The presence of cybersickness often concerns investigators when using fully immersive technologies. Yet, it has been found, not only for stroke patients but also for older adults, that immersion causes minimal cybersickness (Despoti et al., 2022) and led to a positive attitude toward this intervention (Huygelier et al., 2019; Specht et al., 2021).

In the study by Jaffe et al. (2004), patients had to step over obstacles either real ones for the control group or virtually displayed for the experimental group. Although both groups improved, the VR group demonstrated a greater improvement during fast speed walking, as a gait variable. This could probably be the result of the constant exercise offered by the treadmill and the HMD in contrast to the specific hallway in the control group. The better performance could be also attributed to the different stimuli (auditory and visual) offered by the immersive VR environment. It should be noted that the patients in the VR group had even the ability of a lateral view of their legs. The same positive results were seen when the optic flow was used (Kang et al., 2012). Being able to offer different stimuli by altering the optic flow delivered in the immersive environment can increase neural action, especially in motion-sensitive cortical areas (Rutschmann et al., 2000). A recent case study further assessed the use of evolved augmented reality with the use of an optical see-through head-mounted display (OST-HMD). This provided a wider field of view (43 × 29 degrees) compared to other devices and thus could display more virtual objects in a real-world environment such as real-life obstacles and barriers (like blocks and floor mats) that were used in this specific study (Held et al., 2020). Positive results encourage further investigation of the beneficial effect of such devices in the gait rehabilitation of stroke patients.

It is worth mentioning that although a few studies didn’t demonstrate significant differences between groups in the outcomes assessed, the virtual reality intervention group was able to show a significant difference over time. In the study by Crosbie et al. (2012), although the intervention group didn’t present a significant improvement in Motricity Index for upper limb motor function, it did manage to maintain it at follow-up, whereas the control decreased it to baseline.

The gamification of VR rehabilitation interventions is believed to motivate patients to actively participate with pleasure thus increasing the tasks performed and augmenting their recovery. When participants are more interested, they are more concentrated and more persistent in completing their tasks. Certain benefits in psychological outcomes should always be acknowledged as a positive component, especially in neurological patients who often are faced with a long recovery (Qian et al., 2020). Our results are in agreement with those of previous systematic reviews or narrative ones that have examined semi-immersive VR interventions (Yates et al., 2016; Porras et al., 2018; Rutkowski et al., 2020) and with a scoping review that assessed the application of HMD in adult physical rehabilitation (Saldana et al., 2020). The authors did state that the use of HMD as a low-cost, portable tool seems to have additional benefits, but the generalization of the findings is yet to be discussed due to the relatively low level of evidence and the small number of participants (Saldana et al., 2020). Another key aspect noted also by Porras et al. (2018) is the additional benefits of incorporating VR in conventional rehabilitation, such as motivation and engagement.

A few limitations should be noted. One of them is that the population included in most of the studies was small ranging from 8 to 65 stroke patients. Furthermore, patients were included not only immediately after the incidence of the stroke but even being at a more chronic state without determining the exact time that has passed. Time is a key component in the functional rehabilitation of stroke patients as improvements are diminished after a few months. Another important information that is not being shared in most of the studies is the type of stroke: an ischemic or hemorrhagic one. Often, patients from both types were included.

The VR is a continuously developing technology that could offer additional stimulus to traditional rehabilitation strategies augmenting the whole process. As benefits were seen both in upper and lower rehabilitation, the combination should be considered in future studies. Additionally, future studies should be aimed at exploring the use of such technologies in a clinical environment and possibly incorporating both physical and cognitive interventions. Rehabilitation also needs to target sensory deficits, and recent studies have suggested VR’s potential to improve the sensory area. Whether employing sensory and physical tasks in a combined way during VR rehabilitation to further benefit physical components remains to be decided (Tinga et al., 2016).

## Data availability statement

The original contributions presented in the study are included in the article/supplementary material, further inquiries can be directed to the corresponding author.

## Author contributions

IP carried out the literature search, summarized the results, wrote the manuscript, and prepared the tables and figures. ND and EK interpreted the results and supervised the process. AD and DT carried out the literature research and appraised the articles. NL, GR, and AP interpreted the results and prepared the tables. SN appraised the articles and supervised the process. All authors contributed to the article and approved the submitted version.
